# Oral Signs and HLA-DQB1∗02 Haplotypes in the Celiac Paediatric Patient: A Preliminary Study

**DOI:** 10.1155/2013/389590

**Published:** 2013-10-03

**Authors:** M. Erriu, G. M. Abbate, F. M. G. Pili, F. Novara, G. Orrù, C. Montaldo, V. Piras, L. Levrini

**Affiliations:** ^1^Department of Surgical Sciences, Cagliari University, Via Binaghi 4, 09121 Cagliari, Italy; ^2^Faculty of Medicine, School of Oral Hygiene, University of Insubria, 21100 Varese, Italy

## Abstract

Celiac disease (CD) diagnosis can be extremely challenging in the case of atypical patterns. In this context, oral signs seem to play a decisive role in arousing suspicion of these forms of the disease. At the same time, the different expressions of the HLA-DQB1∗02 allele apparently seem to facilitate the interpretation of signs and highlighted symptoms. The aim of this work was to verify whether it is possible to identify a correlation between the development of oral signs and different DQ2 haplotypes in celiac pediatric patients. 44 celiac patients with a medium age of 9.9 were studied. Oral examinations were performed in order to identify recurrent aphthous stomatitis (RAS) and dental enamel defects (DED). The diagnosis of DED resulted as being related to allele expression (*P* value = 0.042) while it was impossible to find a similar correlation with RAS. When both oral signs were considered, there was an increase in correlation with HLA-DQB1∗02 expression (*P* value = 0.018). The obtained results identified both the fundamental role that dentists can play in early diagnosis of CD, as well as the possible role of HLA haplotype analysis in arousing suspicion of atypical forms of the disease.

## 1. Introduction

Celiac disease (CD) is a complex pathologic condition involving the small intestine mucosa resulting from intolerance to gluten assumption [1]. More specifically, it is considered a genetic and autoimmune condition that can affect patients of any age and gender with a great variability of symptoms and clinical signs. Although the final diagnosis of CD is always based on a biopsy with the detection of severe villous atrophy coupled with crypt hyperplasia, the diagnostic pathway leading to this conclusion can often be long and winding [1, 2]. This pathology, initially considered as typical of the European population, is nowadays distributed worldwide. Epidemiological analysis has reported a prevalence of celiac disease as varying greatly between the western and the eastern parts of the world, so that CD is today considered as the most common genetic disorder in the west with a prevalence of 1%, while it is relatively unknown in Asian countries [1, 3]. This irregular distribution in different countries has been related to genetic and alimentary factors. CD is actually an intestinal enteropathy whose symptoms are determined by the ingestion of gliadin in genetically predisposed patients [1]. Gliadin is a prolamin, a class of peptides highly resistant to gastrointestinal enzymes, which causes histological changes in the small intestine mucosa of the celiac patient, leading to a malabsorption syndrome. This abnormal response of the intestinal mucosa is linked to a specific genetic predisposition; almost all the patients affected by CD carry a HLA-DQ2 molecule which is highly frequent in the west and absent in Asian countries. Infections represent another environmental factor identified in the understanding of the CD pathogenesis: the presence of different virus has been described by several authors as a possible cause of pathology development after gluten introduction in the diet [1, 4, 5].

The pathology can be clinically distinguished in different forms, namely, classical, atypical, subclinical, and latent. The classical form is characterized by the typical gastroenterological signs related to the response of the intestinal mucosa after exposure to gliadin. Atypical forms are described with several symptoms and signs in various districts. The oral cavity is an area which is highly affected by extraintestinal signs of the celiac disease, so that lesions in the oral mucosa or defects in dental enamel may often be the only presenting features of the atypical pattern [6–9]. The HLA haplotype was shown to have a strong effect on the distribution of typical and atypical signs. A previous work by Erriu et al. in 2011 related the distribution of recurrent aphthous stomatitis (RAS) and dental enamel defects (DED) in the oral cavity, in both child and adult celiac patients, to the presence or absence of the HLA-DQB1*02 allele [10]. Enamel defects in children were also analysed in another study in 2010 by Majorana et al., who reported the absence of correlation between DED formation and the expression of HLA-DR and -DQ alleles [11].

Based on the correlation detected in the previous 2011 paper between the HLA-DQB1*02 haplotype and oral signs in patients of all ages, the aim of this work was to verify whether the same evidence can be confirmed in pediatric patients.

## 2. Material and Methods

### 2.1. Subjects

44 celiac patients (16 males and 28 females) were analysed, with a medium age of 9.9 years (range 6–16 years), all typed for anti-gluten antibodies (AGA, both IgA and IgG) and the anti-endomysial antibody (EMA). The pathology had been diagnosed for each patient after small bowel biopsy with positivity for enteropathy with Marsh type 3 villous atrophy. Patients with type 1 or 2 intestinal damage were excluded. Furthermore, all patients responded to a gluten-free diet, as evaluated during further regular annual followups. Anamnestic and diagnostic case studies were compiled for each patient to indicate the past or current presence of celiac oral signs, such as RAS and DED [9, 12, 13]. DED were graded from 0 to IV according to Aine's classification [14–16] while RAS linked to CD was determined by the investigation of past experience of aphthous lesions, described by the patients as the contemporaneous presence of one or more ulcers recurring at least twice a month in the period preceding the gluten-free diet and which were not reported 1 month after the start of the diet. The presence of these lesions was verified by evaluating the medical records of the patient's first admission.

An agreement form explaining the aim and the characteristics of the study was read and signed by the participants' parents, and an identifying code was assigned to each subject according to Italian privacy laws. The research was conducted in accordance with Declaration of Helsinki research ethics. Oral brushing was subsequently carried out on all patients, obtaining a sample for DNA extraction allowing the determination of the HLA-DQB1 haplotype using a conventional polymerase chain reaction (PCR) method [17].

All clinical data and samples were obtained from routine testing and visits carried out at the hospital where CD diagnosis had been performed. No new visits or sampling were carried out on patients in order to perform this study.

### 2.2. PCR Technique and Determination of HLA-DQB1 Genotype

The kit constituted of a preformed MIX, and eight couples of primers were used in order to perform the molecular analysis. Positivity or negativity of amplification for each couple allowed the HLA-DQB1 genotype to be established [9, 12, 13]. This set of primers can positively identify the HLA-DQB1 alleles corresponding to the serologically defined series HLA-DQ2, DQ3, DQ4, DQ5, DQ6, DQ7, DQ8, and DQ9; thus, all combinations of DQB1 can be readily identified. DQ4, DQ5, and DQ6 were uniquely identified, whereas DQ2 specificity was amplified by three primer mixes, DQ7 and DQ9 specificities were amplified by two primer mixes, and DQ3 and DQ8 specificities were amplified by four primer mixes.

On the contrary, on examining the eight primer mixes with the corresponding amplified DQB1 alleles, the first primer mix amplified allele group DQB1*05, the second amplified allele group DQB1*06, the third, the fourth, and the sixth amplified allele group DQB1*02, the fourth, the fifth, the sixth, and the seventh amplified allele group DQB1*03, and the eighth amplified allele group DQB1*04.

For all the alleles, the reaction was performed in 10.08 *μ*L reaction volumes using the mixture according to the manufacturer's instructions. The mixture contained 3 *μ*L of master mix, 5 *μ*L of DNAsi-RNAsi free water, 0.08 *μ*L of Taq polymerase, and 2 *μ*L of DNA suspension, and this was put into a tube containing the lyophilized primer pair. The thermocycler profile was performed as follows: an initial denaturation at 94°C for 2 min, 10 cycles consisting of 94°C for 10 sec and 65°C for 1 min, and finally 20 cycles consisting of 94°C for 10 sec, 61°C for 1 min, and 72°C for 30 sec. PCR products were analysed by electrophoresis on an agarose gel.

### 2.3. Statistical Analysis

Descriptive statistics, Fisher's exact test, and binary logistic regression analysis were performed. To test the relation between the HLA haplotype and the presence of oral signs, a logit regression model was used where the outcome variable represents the presence (or the absence) of the specific oral sign to be tested (DED, RAS, or both). A variable was then coded with values 0, 1, or 2 if the individual carried no, one, or two copies of the HLA-DQB1*02 allele, respectively. Values of *P* < 0.05 were considered as significant.

## 3. Results

**Figure 1:**
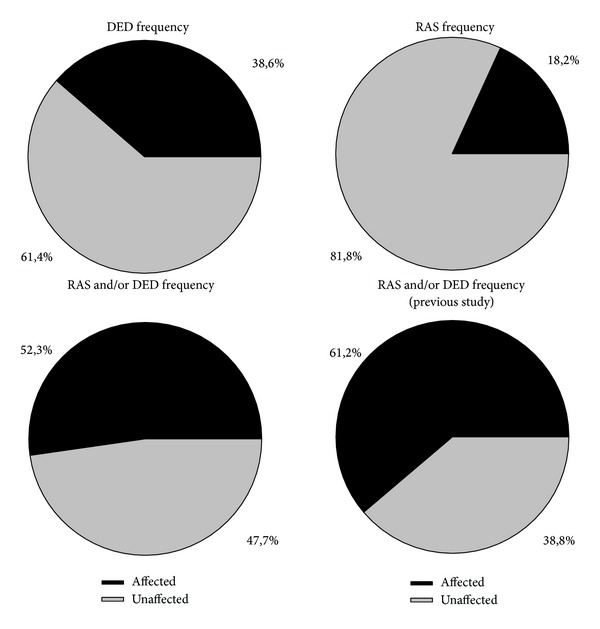


According to the clinical evaluation, the result of the patients affected by RAS was 18.2% (8 persons), while a DED was diagnosed for 38.6% of the patients (17 persons), with 52.3% (23 persons) presenting one or more oral signs (Figure 1). 

HLA-DQB1*02 distribution showed similarities with the previous work by Erriu et al. [10] (Table 1, Figure 1). The percentage of patients carrying two copies of the alleles was 38.6%, and 40.9% showed heterozygosis while only 20.5% did not carry the allele. 

**Figure 2:**
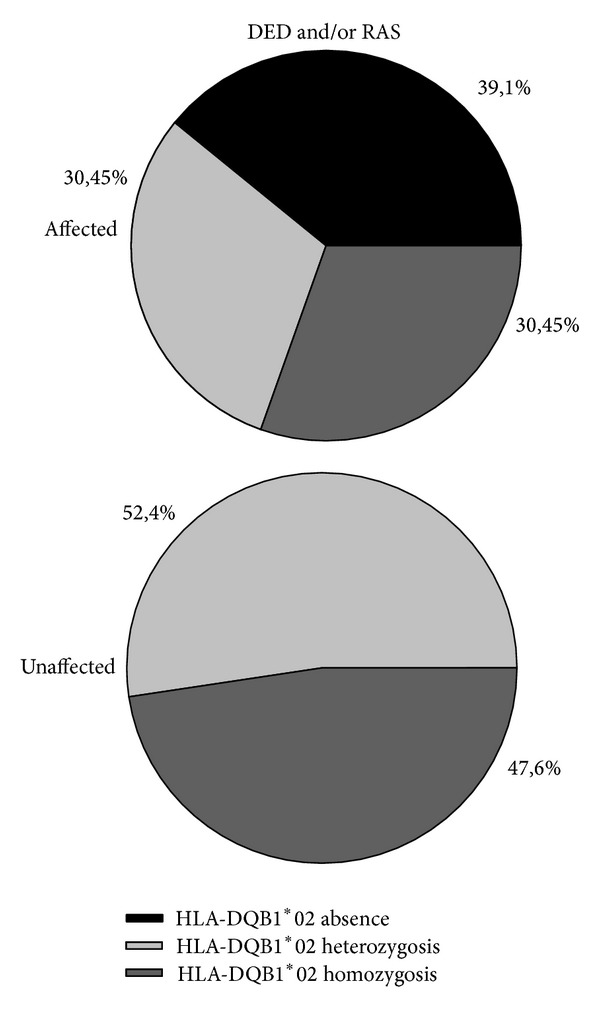


DED diagnosis resulted as being related to the presence or absence of the allele (*P* value = 0.042). On the contrary, it was not possible to find a similar correlation with RAS (*P* value = 0.084). When considering both oral signs, correlation with HLA-DQB1*02 expression increased with a highly significant *P* value (*P* value = 0.018) (Table 2, Figure 2).

## 4. Discussion

Clinical manifestations of CD are variable, depending on the form of the disease. Classical CD is normally diagnosed at a young age, according to the easy recognition of the characteristic signs and symptoms. On the contrary, atypical forms can, in some cases, be hard to detect, making early diagnosis difficult to perform [18]. The awareness of the complications of the late diagnosis of celiac disease makes it necessary to establish guidelines allowing the clinician to suspect the disease early, even in the case of atypical forms. For this reason, extraintestinal symptoms have recently assumed increasing importance in order to arouse suspicion of CD. Oral signs have been described by several authors as diagnostic elements of frequent detection in the course of atypical disease [19]. DED and RAS frequency appears to be variable from study to study, in relation to the age of the patients, the geographical area, and environmental factors. A study by Bucci et al. in 2006 looked for a difference in the distribution of DED and RAS in 72 celiac patients compared with 162 healthy subjects. They described a prevalence of 20% of DED in celiac patients (against the 5.6% of the controls) while no statistical differences were found for RAS. In this study, 33.3% of the celiac patients showed oral ulcers against 23.4% of the controls [20]. In 2010, Costacurta et al. reported a frequency of 33.3% of DED and 8.3% of RAS in an Italian population of 300 celiac patients with a mean age of 8.16 years compared with a cohort of 300 healthy subjects [21]. In this study the authors described a statistically relevant difference between the two groups. A review published in 2011 by Rashid et al. described the prevalence of DED in patients with permanent teeth, as ranging between 9.5% and 95.9% (mean 51.1%) [9, 22]. In the same study, RAS frequency was based on a Canadian study which described patients with this sign, aged less than 16 years old, in 16% of the cases, while adult patients had reported recurrent oral ulcers in 26% of the cases [23, 24]. In 2008 Campisi et al. analysed the frequency of RAS in a group of 269 Italian celiac patients aged from 3 to 17 compared with a control group of 575 clinically healthy subjects. Their analysis identified a RAS frequency of 22.7% in the patients with CD against the 7.1% of the control group [25]. In 2011 Erriu et al. described the frequency of DED and RAS in a population of 98 celiac patients. In this work DED were identified in 28.6% of the cases and RAS in 38.8% of the patients [10]. In 2012 Yaşar et al. studied the prevalence of RAS in a cohort of 82 patients in Istanbul and identified the patients affected by RAS before esophagogastroduodenoscopy. Their results showed that the prevalence of CD in the RAS population did not significantly differ from that of the unaffected matched population [26]. In the same year El-Hodhod et al. performed a similar study, evaluating 140 Egyptian patients with DED, aged 4–12, while the control group was represented by 720 healthy children. From their analysis, celiac disease was diagnosed in 25% of the patients with DED, against the 0.97% of the control group [27].

The prevalence of oral signs in this paper resulted as being similar to that highlighted in the literature. In fact, DED were identified in 38.6% of the cases while RAS was observed in 18.2% of the patients. In comparison with the previous work performed in 2011, it was possible to identify a reduction of the cases of RAS [10]. This could be explained by the fact that some cases of RAS could have passed unobserved due to the gluten-free diet started in early childhood. 

This high variability in the prevalence of the oral signs was analysed in 2011 in the study by Erriu et al. which showed a statistically significant correlation between oral manifestations and HLA expression [10]. In the present study this correlation resulted statistically confirmed in the younger patients. The HLA-DQB1*02 allele has a fundamental influence on the pathogenesis of CD. The study by Jores et al. showed a positive association between an increasing frequency of DQB1*0201 allele homozygosis and the severity of intestinal damage [28]. On the contrary, an only partially explained negative association does seem to exist in the oral cavity. The hypothesis elaborated in 2011 has not yet been adequately detailed in order to reach a conclusive observation. Both the histological diversity of oral and intestinal mucosa, such as the different oral bacterial flora, as well as a reaction related to additional stimuli typical of the oral cavity, could all be possible responses to the phenomena described [10]. 

## 5. Conclusions

The need of an early diagnosis for all the forms of CD is still an open challenge. This study has shown how the role of dentists in identifying atypical patterns could be fundamental. In particular, the presence of oral signs could be very important for detection in the youngest patients, avoiding the several complications related to CD in adulthood. 

## Figures and Tables

**Table 1:** 

	HLA-DQB1*02 homozygosis	HLA-DQB1*02 heterozygosis	HLA-DQB1*02 absence	Trials
Previous study [10]	33	47	18	98
Current study	17	18	9	44
*P* value	0.574	0.471	0.819	

**Table 2:** 

Copies of HLA-DQB1*02	*N* patients	*N* affected	*N* unaffected	Affected %	Unaffected in %	OR (CI 95%) *P* value
DED						
0	9	8	1	88.9%	11.1%	0.40 (0.17–0.97) **0.042**
1	18	3	15	16.7%	83.3%	
2	17	6	11	35.3%	64.7%	

RAS						
0	9	3	6	33.3%	66.7%	0.38 (0.13–1.14) **0.084**
1	18	4	14	22.2%	77.8%	
2	17	1	16	5.9%	94.1%	

DED and/or RAS						
0	9	9	0	100.0%	0.0%	0.33 (0.13–0.82) **0.018**
1	18	7	11	38.9%	61.1%	
2	17	7	10	41.2%	58.8%	
